# Atrioventricular Block Necessitating Chronic Ventricular Pacing After Tricuspid Valve Surgery in Patients With a Systemic Right Ventricle: Long-Term Follow-Up

**DOI:** 10.3389/fcvm.2022.870459

**Published:** 2022-05-10

**Authors:** Marieke Nederend, Monique R. M. Jongbloed, Philippine Kiès, Hubert W. Vliegen, Berto J. Bouma, Madelien V. Regeer, Dave R. Koolbergen, Mark G. Hazekamp, Martin J. Schalij, Anastasia D. Egorova

**Affiliations:** ^1^CAHAL, Center for Congenital Heart Disease Amsterdam Leiden, Leiden University Medical Center, Leiden, Netherlands; ^2^Department of Cardiology, Leiden University Medical Center, Leiden, Netherlands; ^3^Department of Anatomy and Embryology, Leiden University Medical Center, Leiden, Netherlands; ^4^CAHAL, Center for Congenital Heart Disease Amsterdam Leiden, Amsterdam University Medical Center, Amsterdam, Netherlands; ^5^Department of Cardiothoracic Surgery, Leiden University Medical Center, Leiden, Netherlands; ^6^Department of Cardiothoracic Surgery, Academic Medical Center, Amsterdam, Netherlands

**Keywords:** transposition of the great arteries (TGA), systemic right ventricle, heart failure, congenital heart disease, tricuspid valve surgery, pacemaker, cardiac resynchronization therapy

## Abstract

**Background:**

Patients with transposition of the great arteries (TGA) after an atrial switch or congenitally corrected TGA (ccTGA) are prone to systemic right ventricular (sRV) failure. Tricuspid valve (TV) regurgitation aggravates sRV dysfunction. Timely TV surgery stabilizes sRV function, yet the development of atrioventricular (AV)-conduction disturbances in the course of sRV failure can contribute to sRV dysfunction through pacing-induced dyssynchrony. This study aims to explore the incidence, timing, and functional consequences of AV-block requiring ventricular pacing after TV surgery in patients with sRV.

**Methods:**

Consecutive adolescent and adult patients with an sRV who underwent TV surgery between 1989 and 2020 and followed-up at our center were included in this observational cohort study.

**Results:**

The data of 28 patients (53% female, 57% ccTGA, and a mean age at surgery 38 ± 13 years) were analyzed. The mean follow-up was 9.7 ± 6.8 years. Of the remaining 22 patients at the risk of developing high degree AV-block after TV surgery, 9 (41%) developed an indication for chronic ventricular pacing during follow-up, of which 5 (56%) within 24 months postoperatively (3 prior to hospital discharge). The QRS duration, a surrogate marker for dyssynchrony, was significantly higher in patients with chronic left ventricular pacing than in patients with native AV-conduction (217 ± 24 vs. 116 ± 23 ms, *p* = 0.000), as was the heart failure biomarker NT-pro-BNP [2,746 (1,242–6,879) vs. 495 (355–690) ng/L, *p* = 0.004] and the percentage of patients with ≥1 echocardiographic class of deterioration of systolic sRV function (27 vs. 83%, *p* = 0.001). Of the patients receiving chronic subpulmonary ventricular pacing (*n* = 12), 9 (75%) reached the composite endpoint of progressive heart failure [death, ventricular assist device implantation, or upgrade to cardiac resynchronization therapy (CRT)]. Only 4 (31%) patients with native AV-conduction (*n* = 13) reached this composite endpoint (*p* = 0.027).

**Conclusion:**

Patients with a failing sRV who undergo TV surgery are prone to develop AV-conduction abnormalities, with 41% developing an indication for chronic ventricular pacing during 10 years of follow-up. Patients with chronic subpulmonary ventricular pacing have a significantly longer QRS complex duration, have higher levels of the heart failure biomarker NT-pro-BNP, and are at a higher risk of deterioration of systolic sRV function and progressive heart failure.

## Introduction

Over the past decades, surgical, interventional, and pharmaceutical strategies for patients with congenital heart disease (CHD) have improved with over 90% of patients currently reaching adulthood. This increase in survival, however, often comes at the price of long-term sequelae. A particularly challenging group within the adult patient with CHD population is formed by patients with a systemic right ventricle (sRV) in a biventricular circulation. This group entails patients with transposition of the great arteries (TGA) after atrial switch according to Mustard or Senning and patients with congenitally corrected TGA (ccTGA). In this context, the morphological right ventricle is in the subaortic position, supports the systemic circulation, and is exposed to a chronic pressure overload, ultimately resulting in systolic dysfunction of the sRV (1). Atrioventricular (AV) conduction disturbances and tricuspid valve (TV) regurgitation often aggravate the course of sRV failure in these patients (2–4).

Recent literature reports that up to 10% of the atrial switch patients with TGA have AV conduction disturbances requiring ventricular pacing by the age of 28 years (5). For patients with ccTGA, high degree AV-block rates of 30–50% have been reported by the age of 50 years, with an annual risk of developing a *de novo* AV-block of ~2% (2, 6–8). AV-block necessitating chronic subpulmonary ventricular pacing leads to pacing-induced dyssynchrony, which further worsens the sRV dysfunction and may result in heart failure (9, 10). Cardiac resynchronization therapy (CRT) in selected patients with sRV may have the potential to limit some of the negative pacing-induced effects (9–13). Careful patient evaluation and selection as well as awareness of the anatomical limitations of attaining an optimal lead position are essential in achieving successful CRT (9, 11, 12).

Another commonly encountered complication in the sRV failure cohort is tricuspid valve (TV, the systemic AV valve in this anatomy) regurgitation (2). Ebstein-like valves resulting in primary TV regurgitation (TR) are common in patients with ccTGA (14). Functional TR is frequently encountered, mostly as a consequence of annulus dilatation and leaflet restriction due to the progressively dilating sRV (15). Severe TR is reported in 8% of patients with TGA after an atrial switch (5). TR itself results in volume overload and further contributes to sRV dysfunction. Timely TV surgery can stabilize sRV function (2, 15). However, TV surgery in non-congenital cardiothoracic surgery patients is an independent risk for AV-block and ventricular pacing, potentially reducing the beneficial effects of TV surgery (16, 17). To date, no data are available on AV-conduction abnormalities and ventricular pacing strategies in patients with sRV who underwent TV surgery.

This study aims to explore the incidence, timing, and functional consequences of a high degree AV-conduction block requiring chronic ventricular pacing after TV surgery in patients with sRV.

## Methods

### Design and Inclusion/Exclusion Criteria

This two-center, observational, retrospective cohort study was performed at the departments of Cardiology of the Leiden University Medical Center and Amsterdam University Medical Center, united in the Center for Congenital Heart Disease Amsterdam Leiden (CAHAL). Consecutive adolescent and adult patients (≥16 years of age) with an sRV in a biventricular circulation who underwent TV surgery at CAHAL between 1989 and 2020 were included for analysis. Patients with perioperative in-hospital mortality were excluded due to a lack of follow-up (*n* = 3).

### Data Collection and Follow-Up

Clinical and demographic data were collected from electronic patient records. All the reported follow-up visits and investigations were performed as the part of routine clinical care. Data were collected at baseline (latest outpatient clinic visit prior to the TV surgery), postoperatively prior to hospital discharge, and at the last outpatient clinic visit before reaching the composite clinical endpoint of progressive heart failure (CRT, ventricular assist device (VAD) implantation/heart transplantation, or death) or the most recent follow-up available. The closing date for follow-up was March 2021.

During the study, the data collected were medical history, complaints, pharmacological therapy, physical examination, cardiac implantable electronic device interrogation findings, electrocardiography (ECG), echocardiography, and laboratory investigations. For TV surgery, surgical technique [TV annuloplasty (TVP) or TV replacement (TVR)], and concomitant procedures and complications were documented. Data on vital status and cause of death were obtained.

Serial echocardiograms were performed with commercially available ultrasound systems and were analyzed offline in Echo PAC (GE Medical Systems, USA). Echocardiographic parameters were assessed and measured offline by two independent cardiologists with expertise in congenital imaging.

### Outcomes

**Figure 1:**
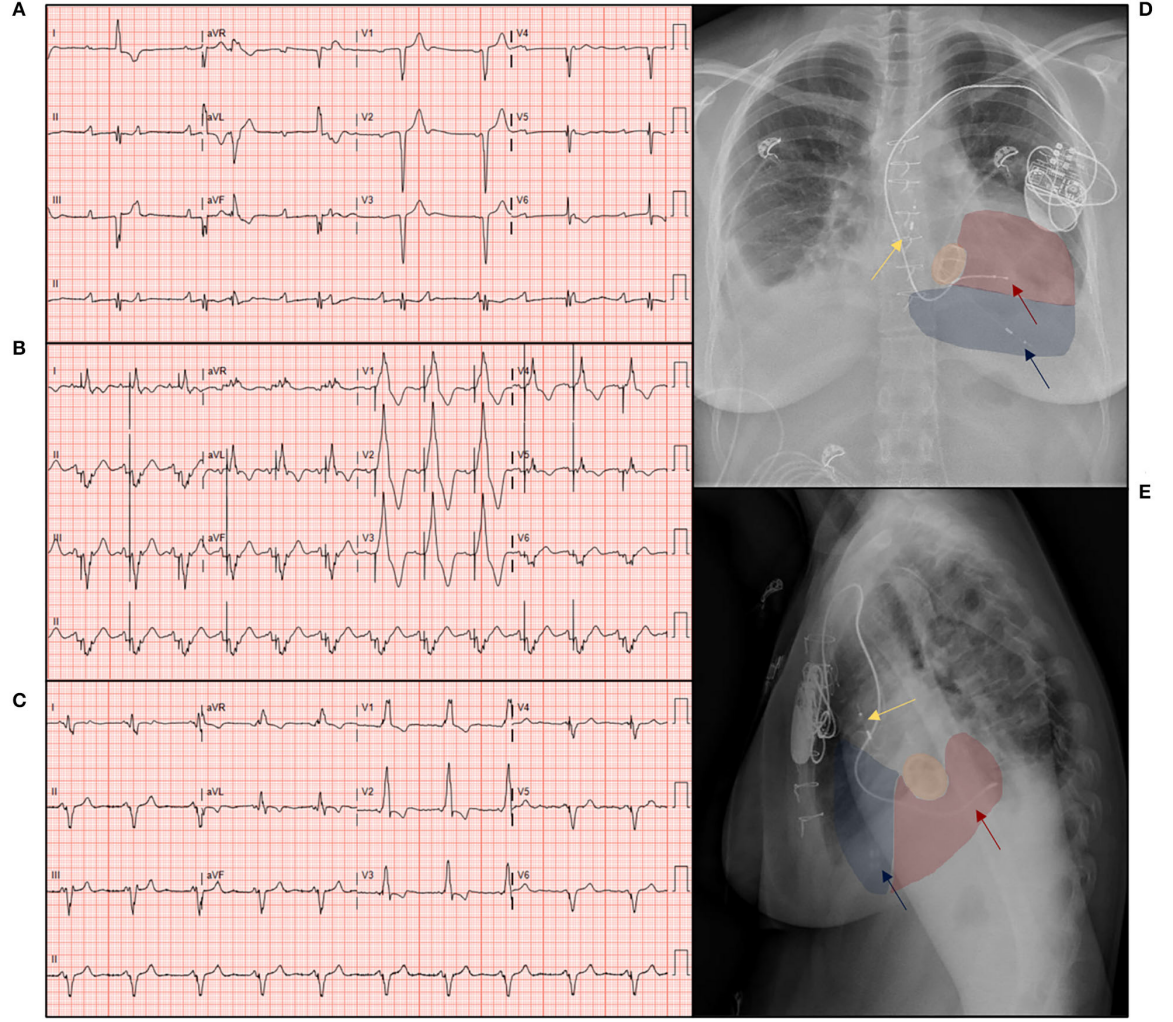
The 12-lead ECG recordings and chest X-rays of a patient with congenitally corrected transposition of the great arteries, illustrating the effects of subpulmonary ventricular pacing, and cardiac resynchronization therapy on an electrocardiogram (ECG). The 12-lead ECG with **(A)** sinus rhythm and a total atrioventricular (AV)-block with ventricular/junctional escape rate of 48/min; **(B)** sequential subpulmonary left ventricular pacing of 75/min, note the broad paced QRS complex of 245 ms; **(C)** biventricular pacing after upgrade to a cardiac resynchronization therapy (CRT), note the reduction of QRS duration to 137 ms, illustrative of the electrical contribution of the systemic right ventricular (sRV) activation. **(D)** Postero-anterior, **(E)** lateral chest X-ray showing the pacing lead in the coronary sinus (red arrow) in the systemic right ventricle (red), atrial lead in the right atrium (yellow arrow), and transvenous pacing and ICD leads (blue arrow) in the subpulmonary left ventricle (LV) (blue), note the mechanical tricuspid valve *in situ* (orange).

The primary outcome was the incidence and timing of high degree AV-block necessitating ventricular pacing. Chronic ventricular pacing was defined as > 40% subpulmonary left ventricular (LV) pacing, in accordance with the current ESC guidelines (2). Secondary outcomes consisted of clinical outcomes, such as the composite clinical endpoint of progressive heart failure (all-cause mortality, VAD implantation/heart transplantation, and implantation/upgrade to CRT). A representative example of the electrocardiographic effects of LV and CRT pacing modalities in a ccTGA patient with a high-degree AV-block is shown in Figure 1. The first event per patient was taken for analysis, and functional clinical parameters [such as, New York heart association (NYHA) functional class, pharmacological therapy, ECG parameters, laboratory findings, including biomarker N-terminal-pro hormone B-type natriuretic peptide (NT pro-BNP), and echocardiographic parameters] were collected at that point. All patient data were described, as well as a comparative analysis between patients with chronic LV pacing and patients with native AV-conduction. Patients with CRT prior to or concomitant during TV surgery were excluded from the comparison between clinical outcomes at the last follow-up and the incidence of the composite endpoint.

### Ethics Statement

All tests and procedures performed involving human participants were in accordance with the ethical standards of the institutional and/or national research committee and with the 2013 Helsinki declaration or comparable ethical standards. Appropriate local scientific board approval was obtained and the need for written informed consent was waived by the institutional medical ethical board. All patients provided consent for their data to be registered and published.

### Statistical Analysis

All statistical analyses were performed in IBM SPSS version 25 and the cumulative incidence curve was performed in R version 3.6.3 (R Foundation for Statistical Computing, Vienna, Austria). Normally distributed continuous data are displayed as mean ± standard deviation (SD) and non-normally distributed continuous data are displayed as median and the first and third interquartiles [IQ1 and IQ3]. Proportions are displayed as numbers (percentage, %). The primary analysis focused on the incidence and timing of chronic ventricular pacing. A cumulative incidence curve was generated for pacing dependency after surgery. A comparison between patients with native AV-conduction and chronic ventricular pacing was performed using an unpaired-samples *T*-test or the Mann–Whitney *U*-test, as appropriate. In the case of categories, a McNemar test or the chi-square test was used. For a comparison between the occurrence of the cumulative endpoint of CRT, VAD implantation/heart transplantation, or death, the chi-square test was used. The value of *p* < 0.05 was considered statistically significant.

## Results

### Patient Characteristics

**Figure 2:**
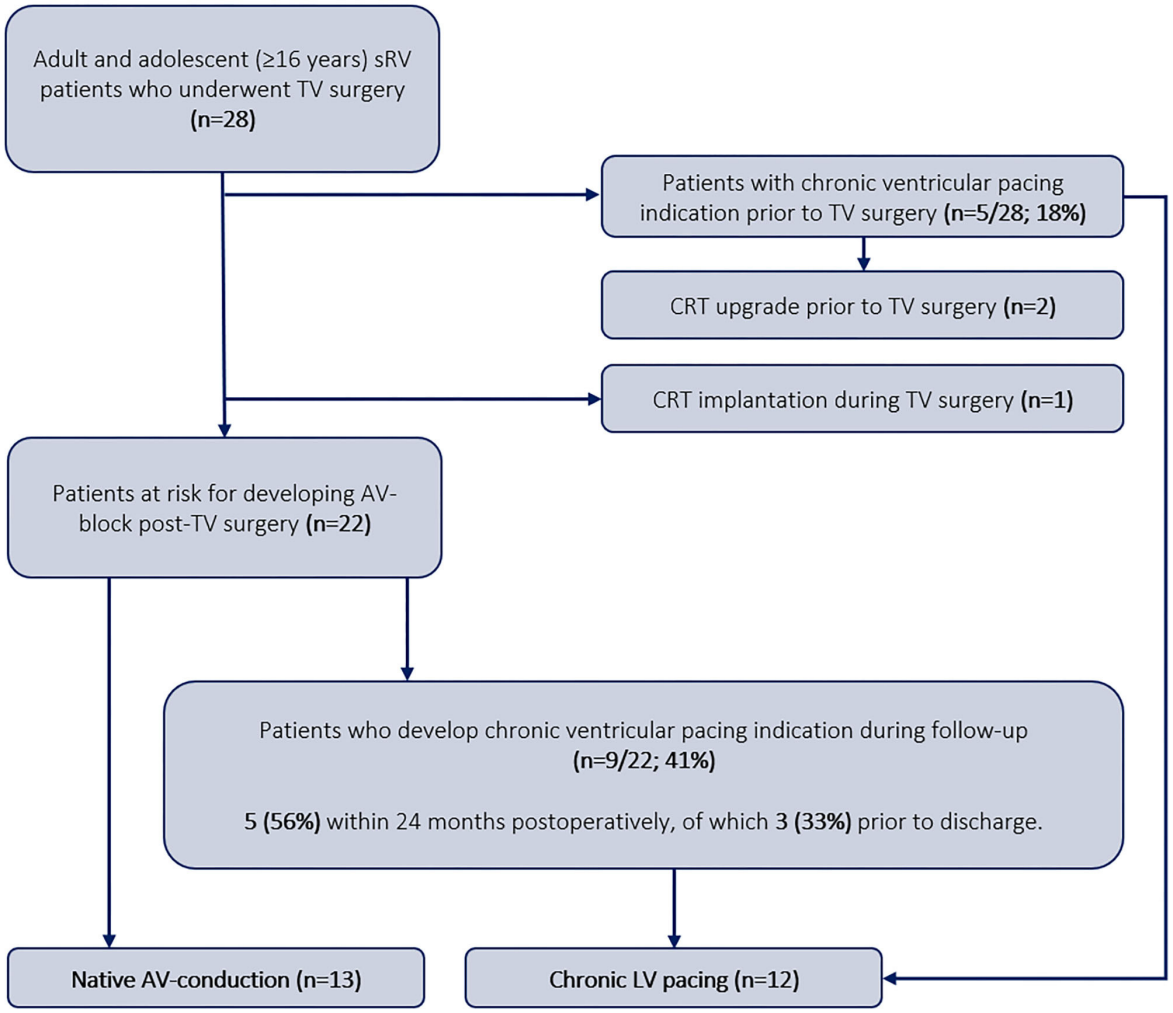
Overview of the study population and the follow-up. sRV, systemic right ventricle; TV, tricuspid valve; CRT, cardiac resynchronization therapy; AV, atrioventricular; LV, subpulmonary left ventricle.

Data from 28 patients were included for analysis (Figure 2). Patient characteristics at baseline are shown in Table 1. Fifteen patients (53%) were women and 16 patients (57%) had ccTGA anatomy. The mean age at the time of TV surgery was 38 ± 13 years and the mean follow-up after surgery was 9.7 ± 6.8 years. Twenty-four patients (86%) used heart failure medication, such as beta blocker, angiotensin-converting enzyme inhibitor, angiotensin receptor blocker or angiotensin receptor-neprilysin inhibitor, mineralocorticoid receptor antagonists, and loop or thiazide diuretics. The majority of patients were in NYHA functional class III-IV (15 patients, 53%). Two (7%) patients had a normal systolic sRV function, 9 (32%) had a mildly reduced sRV function, 14 (50%) moderately reduced, and 1 patient (4%) had a severely reduced sRV function. A total of 11 (39%) patients underwent TVP and 17 (61%) TVR, of which 5 (29%) received a biological and 12 (71%) received a mechanical valve prosthesis. Indications for TV surgery were at least moderate TR or mild TR with poor systolic sRV function. Seven (25%) patients underwent concomitant baffle/conduit replacement or baffle dilatation plasty, and six patients (21%) underwent mitral valve annuloplasty.

**Table 1:** Patient characteristics at baseline and patient characteristics compared between the group that retains native atrioventricular (AV)-conduction and the group that develops high-degree AV-block.

**Patient characteristics at baseline**	**All** **(*n* = 28)**	**Native AV-conduction** **(*n* = 13)**	**Chronic ventricular pacing** **(*n* = 12)**	***P*-value**
Age at surgery (years) (mean ± SD)	38 ± 13	40.5 ± 12.1	38.2 ± 13.1	0.643
Female	15 (54%)	6 (46%)	6 (50%)	0.848
Anatomy				0.821
TGA with atrial switch procedure	12 (43%)	6 (46%)	5 (42%)	
ccTGA	16 (57%)	7 (54%)	7 (58%)	
NYHA functional class				0.429
NYHA I-II	10 (36%)	5 (38%)	5 (42%)	
NYHA III-IV	15 (54%)	8 (62%)	4 (33%)	
Unknown	3 (11%)	0 (0%)	3 (25%)	
**ECG findings**				
Rhythm				0.183
Sinus rhythm	16 (57%)	9 (69%)	5 (42%)	
Atrial fibrillation or flutter	5 (18%)	2 (15%)	2 (17%)	
Atrial rhythm	1 (4%)	0 (0%)	1 (8%)	
Atrial pacing	3 (11%)	1 (8%)	2 (17%)	
PR duration (msec) mean ± SD	189 ± 43	192 ± 24	196 ± 53	0.838
QRS duration (msec) mean ± SD	140 ± 35	134 ± 34	144 ± 35	0.523
**Pharmacological therapy**				
Beta blocker	9 (32%)	5 (39%)	4 (33%)	0.779
ACE-i/ARB/ARNI	19 (68%)	11 (85%)	7 (58%)	0.683
MRA	3 (11%)	2 (15%)	1 (8%)	0.774
Diuretics (loop and thiazide)	12 (43%)	6 (46%)	6 (50%)	0.342
None	4 (14%)	1 (8%)	1 (8%)	0.784
**Echocardiography**				
Qualitative sRV function				0.051
Normal	2 (7%)	0 (0%)	1 (8%)	
Mildly reduced	9 (32%)	2 (15%)	7 (58%)	
Moderately reduced	14 (50%)	9 (69%)	3 (25%)	
Severely reduced	1 (4%)	1 (8%)	0 (0%)	
Unknown	2 (7%)	1 (8%)	1 (8%)	
Tricuspid regurgitation				0.361
Grade II	1 (4%)	0 (0%)	1 (8%)	
Grade III	15 (54%)	6 (46%)	7 (58%)	
Grade IV	10 (36%)	6 (46%)	3 (25%)	
Unknown	2 (7%)	1 (8%)	1 (8%)	
**Surgery**				
Type of tricuspid valve intervention				0.198
TVR	17 (61%)	8 (62%)	7 (58%)	
Biological valve	5 (29%)	4 (50%)	1 (14%)	
Mechanical valve	12 (71%)	4 (50%)	6 (86%)	
TVP	11 (39%)	5 (38%)	5 (42%)	
Concomitant procedures	19 (68%)	9 (69%)	8 (67%)	0.891
AP banding	2 (7%)	0 (0%)	2 (17%)	0.125
Baffle leak closure	4 (14%)	2 (15%)	1 (8%)	0.588
Baffle/conduit replacement or dilatation plasty	7 (25%)	3 (23%)	4 (33%)	0.568
MV annuloplasty	6 (21%)	3 (23%)	2 (17%)	0.689
VSD closure	1 (4%)	0 (0%)	1 (8%)	0.288
CABG	1 (4%)	1 (8%)	0 (0%)	0.327

*Data are expressed as n (%) unless otherwise indicated*.

*SD, standard deviation; TGA, transposition of the great arteries; ccTGA, congenitally corrected TGA; NYHA, New York Heart Association; ECG, electrocardiogram; Q, quartile; P/PM, pacemaker; CRT, cardiac resynchronization therapy; ACE-i, angiotensin-converting enzyme inhibitor; ARB, angiotensin receptor blockers; ARNI, angiotensin receptor-neprilysin inhibitor; MRA, Mineralocorticoid receptor antagonists; sRV, systemic right ventricle; TVR, tricuspid valve replacement; TVP, tricuspid valve annuloplasty; AP, arteria pulmonalis; MV, mitral valve; VSD, ventricular septum defect; CABG, coronary artery bypass graft surgery*.

### Primary Outcome: High Degree AV-Block Requiring Chronic Ventricular Pacing

**Figure 3:**
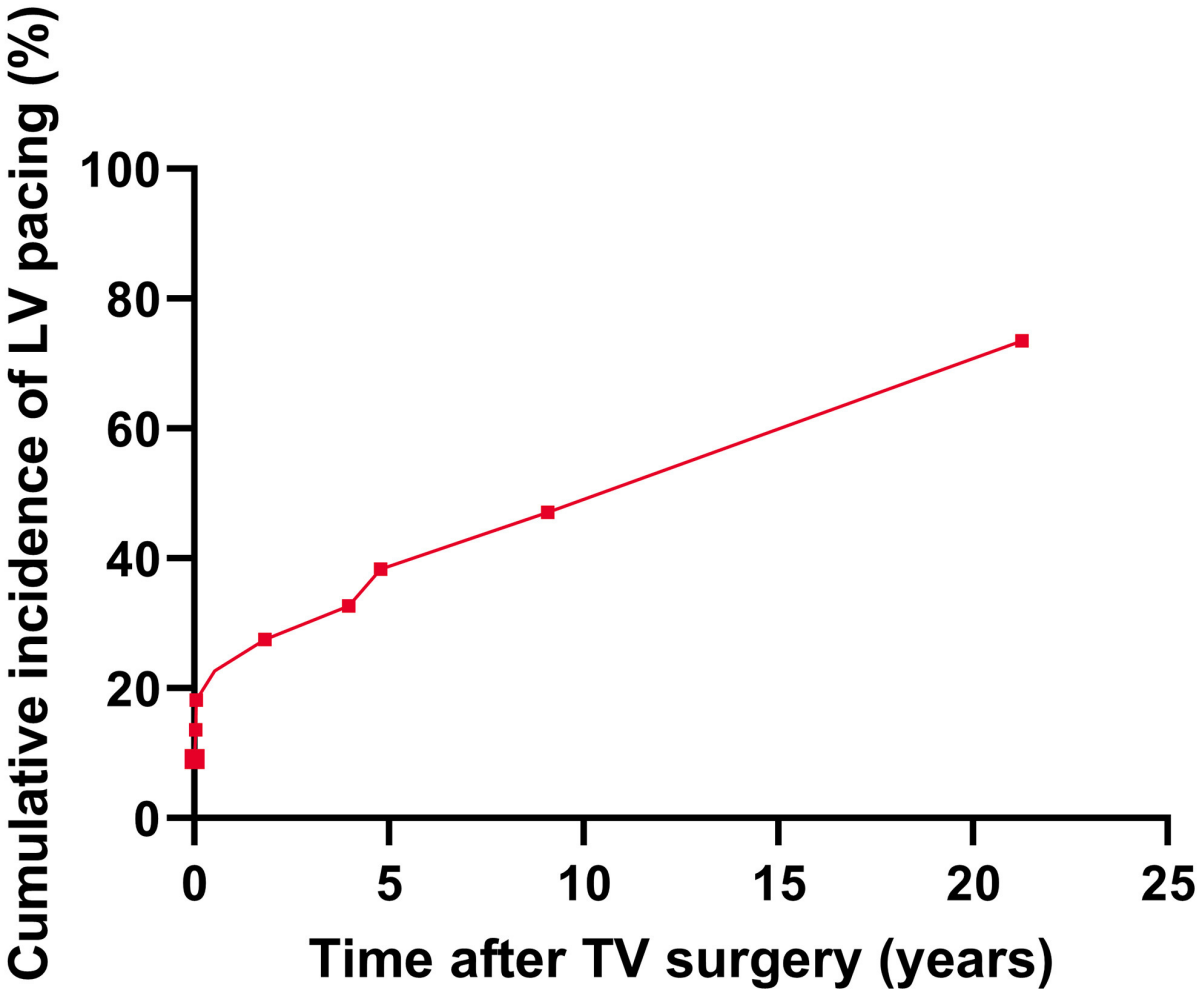
Cumulative incidence curve for chronic LV pacing after TV surgery.

Five (18%) patients received chronic LV pacing preoperatively, of which two had undergone an upgrade to a CRT prior to TV surgery. One patient underwent a concomitant surgical CRT implantation *de novo*. Of the remaining 22 patients at the risk of developing high degree AV-block after TV surgery, 9 (41%) developed an indication for chronic ventricular pacing during follow-up, of which 5 (56%) within 24 months postoperatively (3 prior to hospital discharge) (Figure 2). Of the three patients requiring chronic ventricular pacing before hospital discharge, one patient developed a high degree AV-block during surgery, requiring immediate postoperative pacing. The other two patients developed a high degree AV-block within 48 h after surgery. This resulted in a total incidence of AV-block necessitating chronic ventricular pacing of 50% (14 patients). The cumulative incidence curve for the development of high degree AV-block necessitating chronic ventricular pacing after TV surgery is shown in Figure 3.

There were no significant differences between the group of patients retaining native AV-conduction and those requiring chronic ventricular pacing with regards to baseline demographic characteristics, anatomy, preoperative ECG characteristics, echocardiography, surgical technique, and concomitant procedures or pharmacological therapy (Table 1).

### Secondary Outcomes

During follow-up, 7 (25%) patients died, of which 6 deaths were heart failure-related and one patient died due to aspiration pneumonia. Three (11%) patients underwent VAD implantation as destination therapy due to progressive heart failure and being not eligible for heart transplantation. No patients received heart transplantation. During follow-up, 5 (5/25, 20%) patients received CRT implantation or upgrade due to progressive heart failure. Of all the CRT implantations and upgrades, pre- and post-surgery, four patients (50%) required a hybrid and/or primarily surgical approach. One patient received a transvenous upgrade to CRT, yet successful resynchronization was not achieved due to a suboptimal lead location. Seven (26%) patients required a re-operation, other than for epicardial lead placement, of which three VAD implantations, three TVR procedures (after initial TVP), and one pulmonary valve replacement.

**Figure 4:**
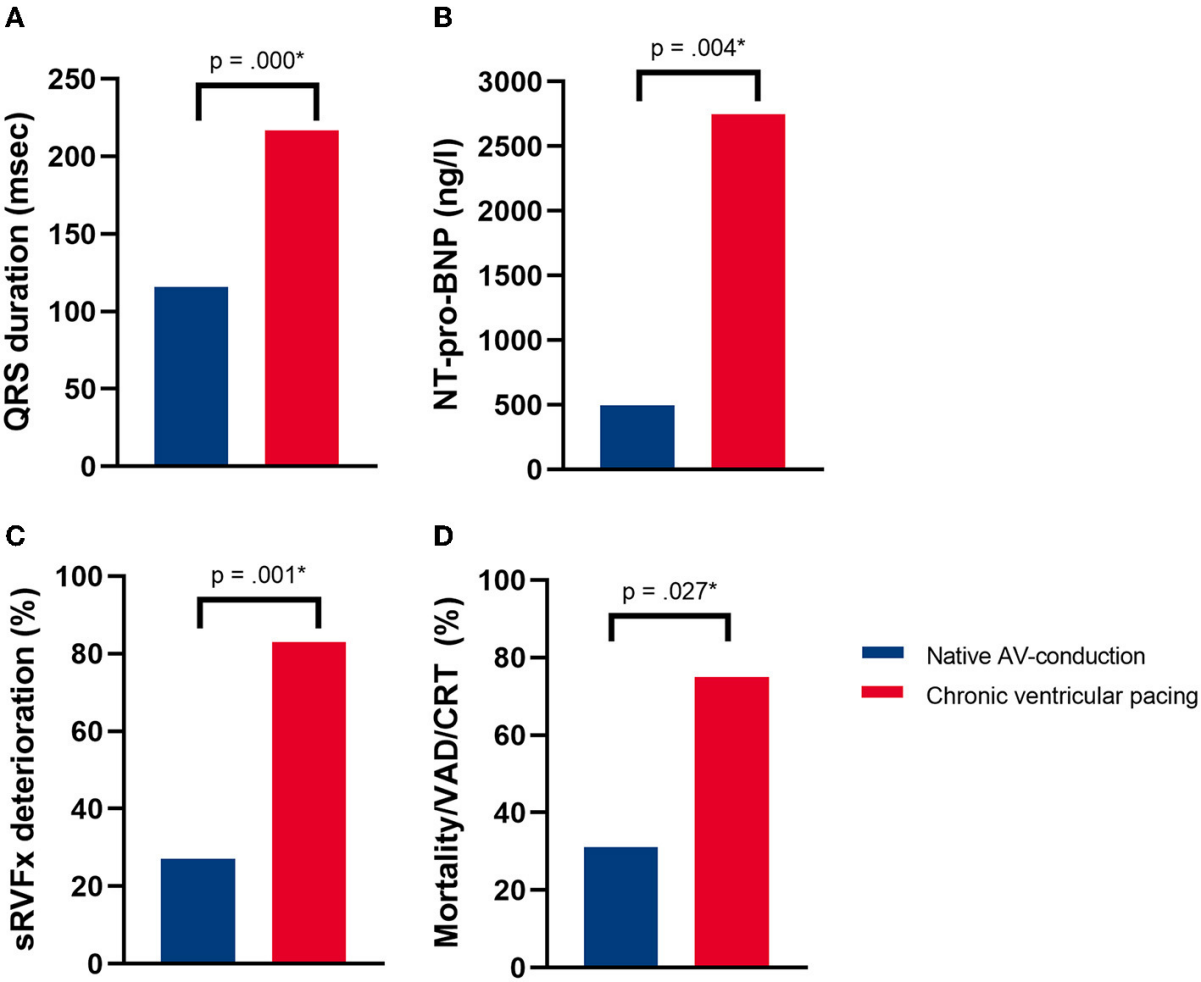
**(A)** Percentage of patients having reached the composite endpoint of mortality, ventricular assist device (VAD) implantation, or upgrade to cardiac resynchronization therapy (CRT) due to progressive heart failure, **(B)** QRS duration (ms), **(C)** N-terminal-pro hormone B-type natriuretic peptide (NT-pro-BNP, ng/L), and **(D)** the percentage of patients with at least one echocardiographic class of deterioration of sRV function at last follow up after TV surgery in patients with native AV-conduction (blue) vs. chronic left ventricular pacing (red). The * symbol indicates statistically significant values.

Clinical outcome data are shown in Table 2. QRS duration was significantly longer in the pacing-dependent group (116 ± 23 vs. 217 ± 24 ms, *p* = 0.000) (Figure 4A). Renal function (estimated glomerular filtration rate, eGFR) was not significantly different between groups (70.5 ± 15.6 vs. 64.2 ± 17.1 ml/min/1.73 m^2^, *p* = 0.449). Figure 4B shows that NT pro-BNP levels were, however, significantly higher in patients with chronic ventricular pacing [495.0 (355.0–690.0) in the group with native AV-conduction vs. 2,746.0 (1,242.0–6,879.0) ng/L among pacing patients, *p* = 0.004]. The global sRV function and percentage of patients with severely reduced sRV function were not significantly different between the groups (Table 2). However, the percentage of patients who had a deterioration of ≥ 1 echocardiographic class of systolic sRV function was higher in patients with chronic ventricular pacing compared with those who retained their native AV-conduction (27 vs. 83%, *p* = 0.001) (Figure 4C). The recurrence of TR was not different among the groups. The NYHA class was not different among the groups.

**Table 2:** Clinical outcomes at last follow-up, patients with cardiac resynchronization therapy before and concomitant TV surgery were excluded from analysis (*n* = 2).

**Clinical outcomes at last follow-up**	**Patients included for analysis (*n* = 25)**	**Native AV-conduction (*n* = 13)**	**Chronic ventricular pacing (*n* = 12)**	***P*-value**
**Clinical and ECG findings**				
NYHA functional class				0.062
NYHA I-II	13 (52%)	9 (69%)	4 (33%)	
NYHA III-IV	10 (40%)	3 (23%)	7 (58%)	
Unknown	2 (8%)	1 (8%)	1 (8%)	
QRS duration (msec)	162 ± 56	116 ± 23	217 ± 24	<0.001^*^
mean ± SD				
**Pharmacological therapy**				
Beta blocker	8 (32%)	3 (23%)	5 (42%)	0.304
ACE-i/ARB/ARNI	19 (76%)	10 (77%)	9 (75%)	0.924
MRA	12 (48%)	5 (39%)	7 (58%)	0.292
Diuretics (loop or thiazide)	13 (52%)	5 (39%)	8 (67%)	0.133
**Laboratory parameters**				
eGFR (ml/min/1.73m^2^)	67.5 ± 16.2	70.5 ± 15.6	64.2 ± 17.1	0.394
mean ± SD				
NT-pro-BNP (ng/l)	846.5 [485.3 – 3332.5]	495.0 [355.0– 690.0]	2746.0 [1242.0 – 6879.0]	0.004^*^
Median [Q1-Q3]				
**Echocardiography**				
Qualitative sRV function				0.186
Mildly reduced	3 (12%)	3 (23%)	0 (0%)	
Moderately reduced	10 (44%)	5 (39%)	5 (42%)	
Severely reduced	10 (44%)	4 (31%)	6 (50%)	0.263
Missing	2 (8%)	1 (8%)	1 (8%)	
At least one echocardiographic class of deterioration of sRV function	13 (52%)	3 (27%)	10 (83%)	0.001^*^
Tricuspid regurgitation				0.816
Grade I or less	16 (64%)	9 (69%)	7 (58%)	
Grade II	5 (20%)	2 (15%)	3 (25%)	
Grade III	2 (8%)	1 (8%)	1 (8%)	
Missing	2 (8%)	1 (8%)	1 (8%)	
**Composite endpoint**				
Composite endpoint	13 (52%)	4 (31%)	9 (75%)	0.027^*^
CRT	5 (20%)	1 (7%)	4 (33%)	
VAD	3 (12%)	2 (15%)	1 (8%)	
Death	7 (28%)	1 (7%)	6 (50%)	

*Data are expressed as n (%) unless stated otherwise*.

* *Is statistically significant*.

*CRT, cardiac resynchronization therapy; VAD, ventricular assist device; ECG, electrocardiogram; NYHA, New York Heart Association; sRV, systemic right ventricle; eGFR, estimated glomerular filtration rate; NT-pro-BNP, N-terminal-pro hormone B-type natriuretic peptide; SD, standard deviation; Q, quartile; ACE-i, angiotensin-converting enzyme inhibitor; ARB, angiotensin receptor blockers; ARNI, angiotensin receptor-neprilysin inhibitor; MRA, mineralocorticoid receptor antagonists*.

At the last follow-up, all patients were treated with at least one heart failure drug. Pharmacological therapy did not differ significantly among the groups (Table 2). A total of 8 (32%) patients used a beta-blocker, 19 (76%) patients used angiotensin-converting enzyme inhibitor, angiotensin receptor blocker, or angiotensin receptor-neprilysin inhibitor, 12 (48%) patients used mineralocorticoid receptor antagonists, and/or 13 (52%) patients used to loop or thiazide diuretics.

Four (31%) patients with native AV-conduction reached the composite endpoint of upgrade or implantation of CRT, VAD implantation, or death, as compared with 9 (75%) patients requiring chronic ventricular pacing (*p* = 0.027) (Table 2 and Figure 4D).

## Discussion

The main findings of this study are that (1) the incidence of AV-block necessitating chronic ventricular pacing in patients with sRV who have undergone TV surgery is 41% after 10 years of follow-up, and in 56% of the cases, high AV-conduction block occurs already within the first 24 months postoperatively; (2) the group receiving chronic left ventricular pacing has longer QRS complex duration, higher levels of the heart failure biomarker NT-pro-BNP, and has a higher percentage of patients with at least one echocardiographic class of deterioration of systolic sRV function; and (3) patients with chronic subpulmonary pacing due to AV-block are at a higher risk of progressive heart failure.

### Current Results in Light of Previous Studies

Given the high prevalence of TR requiring surgical intervention and AV-conduction block in the group of patients with sRV failure, it is important to evaluate the incidence and consequences of chronic subpulmonary pacing after TV surgery, as some of its negative sequelae may be prevented by concomitant implantation of an epicardial sRV lead at the time of surgery and timely initiation of CRT. In the current study, we investigated the incidence, timing, and long-term functional consequences of AV-conduction block requiring ventricular pacing after TV surgery in patients with sRV.

#### sRV Dysfunction

Patients with an sRV are prone to sRV dysfunction and failure (2). The morphological right ventricle supports the systemic circulation, yet the right ventricle is not well-adapted to high pressures, leading to ventricular hypertrophy, and later dilatation, dysfunction, and ultimately failure. In patients with ccTGA, TR is known to contribute to the development of sRV dysfunction (3). Timely TV surgery has positive effects on patients with sRV and can stabilize sRV function (3, 15). These positive effects of (timely) surgery, might be counteracted by pacing-induced dyssynchrony due to AV-conduction abnormalities.

Our data showed a high incidence of patients requiring chronic ventricular pacing after TV surgery of 41%, and a total prevalence of 52%. For patients with ccTGA, high degree AV-block rates of 30–50% have been reported by the age of 50 years, with an annual risk of developing a *de novo* AV-block of ~2% (6–8). The cohort described here has a mean age of 38 years and consists for 2/3 of patients with ccTGA, which are more susceptible to AV-conduction abnormalities due to several developmental and pathophysiological considerations (6). The relatively long course of the AV conduction system, the malalignment of the AV septum, fibrotic changes, and the progressive shift of the intraventricular septum toward the morphological LV as a consequence of the pressure overloaded sRV, all contribute to the high prevalence of AV-block in patients with ccTGA (9, 18). In non-congenital patients with often less complex anatomy, TVR is an established independent risk factor for AV-block post-surgery (16, 17). Multiple predictors have been described, e.g., preoperative active endocarditis, the initial postoperative rhythm of <45 beats per min, cross-clamp time of >60 min, and concomitant mitral valve surgery (16, 17). Combined valve surgery is also a known risk factor for post-surgery AV-block in non-congenital patients (19). Six (21%) patients described in this study had concomitant MV surgery, of which 30% developed AV-block requiring concomitant chronic pacing after surgery. Further research is required to elucidate the mechanisms and risk factors underlying the post-operative AV-conduction abnormalities in patients with sRV undergoing TVR.

#### Clinical Outcomes

Chronic pacing-induced dyssynchrony is known to be a contributing factor to sRV dysfunction, and pacing-induced cardiomyopathy rates are higher in adult patients with CHD than in acquired heart disease (9–11, 20). Some even suggest biventricular pacing should always be pursued above single ventricular pacing in patients with ccTGA (10). In the current study, QRS width is significantly longer in patients with chronic left ventricular pacing, which might be a marker of (pacing-induced) dyssynchrony. Yet, little is known about the correlation of ECG findings and dyssynchrony in patients with sRV, which calls for prospective investigations. Due to the historical cohort and technical imaging limitations, the echocardiographic parameters of dyssynchrony could not be thoroughly evaluated. Our results also comparably show that patients with chronic left ventricular pacing have significantly more events at the composite endpoint of progressive heart failure. Although the role of CRT is not well-established in sRV failure, small yet consistent results of CRT in sRV have been carefully optimistic (2, 9–12, 21). Patient selection is key, and contractility measurements derived from Dp/Dt have been shown to have a reasonable predictive value in patients with CHD, where an improvement of Dp/Dt of >15% is correlated with a clinical response to CRT and improvement in ejection fraction up to 12 years (22, 23). These studies reported that detrimental effects might be prevented by CRT, yet CRT is not always possible due to anatomical and technical limitations. Previous studies reported that epicardial leads have good durability (24). Therefore, there might be a potential for epicardial lead placement at the time of TV surgery. Conduction system pacing in CHD, and patients with sRV in particular, also deserves further evaluation (25–27). Cano and colleagues have recently described a cohort of 20 congenital patients, of which seven patients with an sRV (5 with ccTGA and 2 TGA after atrial switch procedure), who have successfully undergone conduction system pacing (25). This observational study reported improvements in the NYHA class and sRV function at 16 months follow-up. Larger multicenter studies are required to elucidate the long-term outcomes of conduction system pacing in this anatomically and technically challenging patient group.

The data show that NT-pro-BNP levels were significantly higher in patients with chronic ventricular pacing. NT-pro-BNP is a widely used biomarker and has been shown to be a surrogate marker for mortality and heart failure in patients with sRV (28). This is supported by significantly higher rates of sRV function deterioration in patients with chronic ventricular pacing. The role of NT-pro-BNP in identifying patients in (sub)clinical phases of heart failure and in prompting for optimizing pacing strategies, remains to the elucidated.

The described cohort had high mortality of 25% at 10 years of follow-up and 52% reached the composite endpoint of mortality, VAD implantation, or progressive heart failure requiring CRT. No patients underwent heart transplantation, reflecting the scarceness of donor hearts in the Netherlands and the poor eligibility of these patients with complex anatomy and numerous thoracotomies (29). Our findings are in line with the previously described cohort reporting late mortality rates of 15% at 6 years of follow-up after TV surgery in patients with sRV (15). Most patients had sRV dysfunction and probably at least some degree of irreversible cardiac remodeling prior to surgery, suggesting that TV surgery might be more beneficial if performed earlier. We observed that significantly more patients with chronic ventricular pacing reached the composite endpoint for heart failure than patients with native AV-conduction. In non-congenital patients, the presence of a permanent pacemaker after TV surgery was not associated with higher mortality or worse clinical outcomes (16, 17, 19). However, in the sRV cohort, the TV is the systemic AV-valve and chronic subpulmonary pacing after TV surgery is therefore principally different from the non-congenital cohorts described. Additionally, the sRV population is particularly prone to systolic dysfunction, and pacing-induced dyssynchrony is more detrimental in this scenario. Woudstra et al. studied a risk model for patients with TGA after an atrial switch (5). A strong predictor for events in this model was severe tricuspid regurgitation, together with age, sRV, and LV dysfunction, ventricular arrhythmia, and age >1 year at repair. Interestingly, the presence of a cardiovascular implantable electronic device was not a definite predictor in the model, yet no analysis was performed to differentiate chronic ventricular pacing from, e.g., only atrial pacing. Of interest, 64% of the patients classified as high risk had pacemakers, calling for further analysis.

### Potential Implications

This study shows the high prevalence of high degree AV-block necessitating chronic ventricular pacing (shortly) after TV surgery. The group of patients with chronic subpulmonary pacing had significantly more events of the composite endpoint of progressive heart failure during follow-up. This might be due to the deterioration of sRV function due to pacing-induced dyssynchrony. As CRT has shown potential to reduce pacing-induced dyssynchrony, concomitant epicardial sRV lead implantation at the time of TV surgery may halt some of the negative pacing-induced effects on sRV dysfunction.

### Study Limitations

This study is retrospective and observational by nature and involves a cohort of 28 patients. The relatively small study population is reflective of the rareness and complexity of the condition and is the largest study on the long-term outcomes after TV surgery in patients with sRV to date. Although the groups with native AV-conduction and chronic subventricular pacing are comparable, some heterogeneity of the study population should be considered when interpreting the results—the anatomy of ccTGA and TGA after atrial switch has important pathophysiological differences, as well as the different surgical strategies that might have evolved over time. As a consequence of the retrospective nature of this study, follow-up is susceptible to information bias and loss to follow-up. Another limitation to this study is the lack of an ideal counterfactual, as progressive sRV dysfunction is intricately related to TR requiring intervention and to AV-conduction abnormalities.

### Future Perspectives

Future research should focus on the mechanisms of AV-block post-surgery in patients with sRV, the correlation among ventricular pacing burden, electrocardiographic, echocardiographic, and invasive markers of dyssynchrony and sRV function, and heart failure progression (post TV surgery). Another future aspiration is to further investigate the therapeutic role of CRT and conduction system pacing in halting the progression of and stimulating reverse remodeling in sRV failure.

## Conclusion

Patients with a failing sRV who undergo TV surgery are prone to develop AV-conduction abnormalities, with 41% developing an indication for chronic ventricular pacing during 10 years of follow-up. Patients with chronic subpulmonary ventricular pacing have a significantly longer QRS complex duration, have higher levels of the heart failure biomarker NT-pro-BNP, and are at a higher risk of deterioration of systolic sRV function and progressive heart failure.

## Data Availability Statement

The original contributions presented in the study are included in the article. Further inquiries can be directed to the corresponding author.

## Ethics Statement

The studies involving human participants were reviewed and approved by Medisch-Ethische Toetsingscommissie Leiden Den Haag Delft (METC LDD). Written informed consent for participation was not required for this study in accordance with the national legislation and the institutional requirements.

## Author Contributions

AE and MJ contributed to study conception and design. MN and MR contributed to data collection. MN contributed to analysis of results. MN and AE contributed to interpretation of results. MN, MJ, PK, HV, BB, MR, DK, MH, MS, and AE contributed to draft manuscript preparation and reviewed the results. All authors approved the final version of the manuscript.

## Conflict of Interest

The authors declare that the research was conducted in the absence of any commercial or financial relationships that could be construed as a potential conflict of interest.

## Publisher's Note

All claims expressed in this article are solely those of the authors and do not necessarily represent those of their affiliated organizations, or those of the publisher, the editors and the reviewers. Any product that may be evaluated in this article, or claim that may be made by its manufacturer, is not guaranteed or endorsed by the publisher.
